# Btn2a2 Regulates ILC2–T Cell Cross Talk in Type 2 Immune Responses

**DOI:** 10.3389/fimmu.2022.757436

**Published:** 2022-01-25

**Authors:** Michael Frech, Yasunori Omata, Angelika Schmalzl, Stefan Wirtz, Leila Taher, Georg Schett, Mario M. Zaiss, Kerstin Sarter

**Affiliations:** ^1^ Department of Internal Medicine 3, Rheumatology and Immunology, Friedrich-Alexander-University Erlangen-Nürnberg (FAU) and Universitätsklinikum Erlangen, Erlangen, Germany; ^2^ Deutsches Zentrum für Immuntherapie (DZI), FriedrichAlexander-University Erlangen-Nürnberg (FAU) and Universitätsklinikum Erlangen, Erlangen, Germany; ^3^ Department of Orthopaedic Surgery, The University of Tokyo, Tokyo, Japan; ^4^ Department of Internal Medicine 1, University of Erlangen-Nuremberg, Erlangen, Germany; ^5^ Institute of Biomedical Informatics, Graz University of Technology, Graz, Austria

**Keywords:** co-stimulation and co-inhibition receptors, ILC2, butyrophilin, helminth infection, type 2 immunity

## Abstract

Innate lymphoid cells (ILC) not only are responsible for shaping the innate immune response but also actively modulate T cell responses. However, the molecular processes regulating ILC-T cell interaction are not yet completely understood. The protein butyrophilin 2a2 (Btn2a2), a co-stimulatory molecule first identified on antigen-presenting cells, has a pivotal role in the maintenance of T cell homeostasis, but the main effector cell and the respective ligands remain elusive. We analyzed the role of Btn2a2 in the ILC-T cell cross talk. We found that the expression of Btn2a2 is upregulated in ILC2 following stimulation with IL-33/IL-25/TSLP. *In vitro* and *in vivo* experiments indicated that lack of Btn2a2 expression on ILC2 resulted in elevated T cell responses. We observed an enhanced proliferation of T cells as well as increased secretion of the type 2 cytokines IL-4/IL-5/IL-13 following cocultures with Btn2a2-deficient ILC2. *In vivo* transfer experiments confirmed the regulatory role of Btn2a2 on ILC2 as Btn2a2-deficient ILC2 induced stronger T cell responses and prevented chronic helminth infections. Taken together, we identified Btn2a2 as a significant player in the regulation of ILC2–T cell interactions.

## Introduction

Innate lymphoid cells (ILC) were shown to play a role in modulating adaptive T cell responses. ILC are involved in modifying the T helper cell (Th) immune response to microbial and allergen exposure and in autoimmune diseases (1, 2). We could previously show that ILC2-derived IL-4/IL-13 and IL-9-induced type 2 immune responses promoted resolution of arthritis (3, 4). In microbial challenge or allergen exposure, many studies reveal that ILC2 and CD4^+^ T cells interact on multiple levels. Mice with reduced ILC2 numbers show impaired type 2 immune responses upon challenge with the parasitic helminth *N. brasiliensis*, asthma-inducing house dust mite (HDM) antigen, or protease-allergen papain (5–7). Addition of unchallenged ILC2 that were not exogenously stimulated with IL-33 to naïve CD4^+^ T cells induces their differentiation into Th2 cells and inhibits differentiation into Th1 cells (8). In line with these findings, type 2 cytokines are not detectable if CD4^+^ T cells are cultured with ILC2 unable to secrete IL-4 (9). Taken together, these studies show that ILC2-derived IL-4 contributes to the induction of Th2 response; however, an IL-4-independent pathway may also exist (6). It is still widely believed that ILC activity is based on their secretion of soluble factors including cytokines. However, ILC also express stimulatory molecules that are crucial for initiating Th2 cell response such as OX40L, ICOS, and ICOS-L (9–11), and type 2 cytokine production is reduced in ILC2–T cell co-cultures in the presence of respective blocking antibodies (9). Therefore, another way that was shown through which ILC can influence CD4^+^ T cell fate is by their ability to serve as antigen-presenting cells (APC). ILC2 process and present antigen on MHCII, and they co-express CD80/86 and induce proliferation of Th2 cells, although to a lesser extent than professional APC (7, 8). In mice infected with *N. brasiliensis*, MHCII expression on ILC2 was enhanced by STAT6 signaling, supporting the concept that Th2 cells and ILC2 can communicate in an antigen-dependent manner (12). ILC2 that express MHCII and CD80/86 have been shown to acquire and process antigen and thereby induce antigen-specific activation and proliferation of T cells (7).

One prominent and widely studied family of proteins modulating T cell responses is the B7 family of ligands and their receptors, which includes both positive (e.g., B7.1, B7.2, ICOS) and negative (e.g., PDL1, B7S3) co-stimulatory molecules. Besides these well-studied molecules, there are newly discovered members, such as the butyrophilin (BTN) and butyrophilin-like (BTNL) superfamily. The BTN and BTNL superfamily has gained importance due to the observation that the BTNL2 molecule can alter T cell responsiveness (13, 14) and the discovery that genetic polymorphisms in BTN molecules are associated with predisposition to inflammatory human diseases (15).

These findings suggested that BTN molecules influence T cell responses *in vivo* and could therefore prevent the excessive immune response in autoimmune conditions and inflammatory disorders. The two members of the BTN family in mice, Btn1a1 and Btn2a2, are involved in inhibition of T cell activation, and secreted soluble Btn2a2 (sBtn2a2) was found to inhibit proliferation of activated CD4^+^ and CD8^+^ T cells and to reduce T cell receptor signaling as shown by reduced Zap70, CD3ϵ, and ERK phosphorylation (16, 17). Cell-surface Btn2a2 protein expression was documented on CD19^+^ B cells, CD11c^+^ splenic DC, and CD11b^+^Ly6Glow peritoneal macrophages (17). Importantly, we previously showed that Btn2a2 expression on hematopoietic cells is responsible for its effects in T cell balance; however, Btn2a2 expression on the “classical” APC like conventional DC, plasmacytoid DC, and B cells was not responsible for this effect (18). In the present study, we now identify Btn2a2 on ILC2 as a critical negative costimulatory molecule to regulate the ILC-T cell cross talk during inflammation.

## Materials and Methods

### Mice

Age- and sex-matched C57BL/6N mice were purchased from Charles River (Germany). Btn2a2^–/–^ mice were generated by the Wellcome Trust Sanger Institute (Cambridge) on a C57BL/6N background and bred in our local animal facility. Mice were co-housed for 2 weeks prior to start of experiments. OTII mice were kindly provided by Prof. Diana Dudziak from Dermatology Department FAU Erlangen-Nuremberg. Ragγc^-/-^ mice were kindly provided by Prof. Chiara Romagnani from Charité Berlin. B6Ptprca-Pep3b/BoyJ Il7rtm1lmx/J (Il7R^–/–^) mice were kindly provided by Prof. Immo Prinz from Medizinische Hochschule Hannover. All mice were housed, and experiments were conducted under specific pathogen-free conditions. All of the protocols for animal experiments were approved by the local ethics authorities of Regierung von Unterfranken.

### *H. polygyrus* Infection

For helminth infections, mice were inoculated with approximately 200 L3 (infective) larvae by oral gavage (kindly provided by Prof. David Vöhringer, Department of Infection Biology, Institute for Medical Microbiology, Immunology and Hygiene, FAU Erlangen-Nuremberg). Feces were collected at the indicated time points, and eggs were counted under an Axiophot microscope (Zeiss). After 12 days (adoptive transfer experiments) or 35 days, mice were sacrificed, and blood, spleens, MLNs, and small intestines were collected and further analyzed as described below. Worm burdens in small intestines were enumerated in dissected tissue as described (19).

### Cell Culture

ILC2 were cultured in DMEM high glucose (Life Technologies) containing 10% FCS, 1% penicillin–streptomycin solution (HyClone), 50 μM 2-mercaptoethanol, 1 mM sodium pyruvate (Life Technologies), non-essential amino acids (Life Technologies) and 20 mM HEPES (pH 7.4), 50 ng/ml of IL-2, IL-7, IL-25, and IL-33, and 20 ng/ml thymic stromal lymphopoietin (TSLP) (all obtained from PeproTech) to activate and expand ILC2 for ~14 days before coculture or adoptive transfer experiments, as initially described by Duerr and colleagues (20). Prior to experiments, *in vitro* expanded ILC2 were assessed for their integrity by flow cytometry. For coculture experiments, CD4^+^ T cells were isolated by negative selection (Stemcell Technologies) and cultured in RPMI (Life Technologies), supplemented with 10% FCS, 1 mM sodium pyruvate (Life Technologies), 1 mM L-Glutamine, 80 µM 2-mercaptoethanol, 20 mM HEPES (pH 7.4), and 1% penicillin–streptomycin solution (HyClone), either alone or together with WT or Btn2a2^–/–^ ILC2 at a 1:1 ratio in the presence of CD3/CD28 Dynabeads™ (Thermo Fisher Scientific) to promote T cell activation. For coculture experiments with CD4^+^ OTII T cells, the OVA peptide was added at a final concentration of 10 µg/ml. After 96 h, cell-free supernatants were frozen at -20°C for later cytokine detection. Cells were resuspended in PBS and analyzed by flow cytometry as described below. Cytokine detection of cell culture supernatants was conducted using LEGENDplex™ (BioLegend), following the manufacturer’s instructions.

### Adoptive Transfer and Immunization

ILC2 were isolated as recently described (21). In brief, donor mice were hydrodynamically injected with 4 µg each of IL-25 and IL-33 vectors to induce ILC2. Three days postinjection (dpi), ILC2 were sort-purified from spleen and mesenteric lymph nodes (MLNs) and *in vitro* expanded as indicated above. OTII transgenic CD4^+^ T cells were purified by negative selection (Stemcell Technologies) according to the manufacturer’s instructions and assessed at ≥95% purity. One day prior to cell transfer, recipient mice were intraperitoneally (i.p.) immunized with 20 µg Ovalbumin (Invivogen) in 200 µl Imject Alum (Thermo Scientific). 2 × 10^6^ CD4^+^ OTII T cells were co-transferred intravenously (i.v.) with 1 × 10^6^ WT or Btn2a2^–/–^ILC2 into 8–14-week-old recipient hosts as indicated. Mice were sacrificed 3 days post adoptive cell transfer, and spleens were harvested for flow cytometric analyses.

### Tissue Preparation and Flow Cytometry

MLNs and spleens were harvested, and single-cell suspension were prepared at necropsy. For intestinal lamina propria lymphocyte preparations, small intestines were isolated and attached fat was thoroughly removed. Luminal contents were flushed out with ice-cold PBS and intestines were cut longitudinally. To remove epithelial cells and intra-epithelial lymphocytes, tissues were cut into small pieces and incubated in dissociation buffer (HBSS, 5% FCS, 2 mM EDTA, 10 mM HEPES (pH 7.4), 1 mM DTT in HBSS without Ca^2+^, Mg^2+^) for 20 min in a shaking incubator at 37°C, twice. Subsequently, residual EDTA was removed by incubation in cold PBS. The lamina propria layer was isolated by digesting the tissue twice with 1 mg/ml Collagenase II (Merck) and 0.1 mg/ml DNase I (Sigma) in HBSS with 10% FCS and 10 mM HEPES (pH 7.4) for 30 min at 37°C. For lung cell isolation, lungs were perfused through the heart with 20 ml PBS. One lobe per mouse was cut into small pieces and digested with 400 μg/ml of Liberase™ in 500 µl for 30 min at 37°C under constant agitation.

For flow cytometric analyses, cells were stained with antibodies to the following markers: anti-CD45.2 (clone 104, BioLegend), anti-TCR Vβ5.1, 5.2 (clone MR9-4, BioLegend), anti-CD45 (clone 30-F11, BioLegend), anti-CD3ϵ (clone 145-2C11, BioLegend), anti-CD4 (clone GK1.5, BioLegend), anti-CD44 (clone IM7, BioLegend), anti-CD62L (clone, MEL-14, BioLegend), anti-CD80 (clone 16-10A1, BioLegend), anti-I-Ab (clone 25-9-17, BioLegend), anti-ICOS (clone C398.4A, BioLegend), anti-KLRG1 (clone 2F1/KLRG1, BioLegend), anti-PD-1 (clone 29F.1A12, BioLegend), anti-CD5 (clone 53-7.3, BioLegend), anti-Gr-1 (cloneRB6-8C5, Thermo Fisher Scientific), anti-OX40L (clone RM134L, BioLegend), anti-CD25 (clone PC61, BioLegend), anti-TCRγδ (clone GL3, BD Biosciences), anti-TCRαβ (clone H57-597, Thermo Fisher Scientific), anti-CD19 (clone 13-0191-85, Thermo Fisher Scientific), anti-CD8a (clone 13-0081-86, Thermo Fisher Scientific), anti-CD11c (clone N418, eBioscience), anti-FcεRIα (clone MAR-1, Thermo Fisher Scientific), anti-CD127 (clone A7R34, BioLegend), and streptavidin (BD Biosciences). For intracellular staining, cells were fixed and permeabilized utilizing the Foxp3 staining buffer set (eBioscience) and stained with anti-GATA3 (clone TWAJ, eBioscience), anti-Tbet (clone 4B10, eBioscience), anti-Tbet (clone 4B10, eBioscience), anti-Eomes (clone Dan11mag, Thermo Fisher Scientific), anti-RORγt (clone Q31-378, BD Biosciences), anti-Foxp3 (clone FJK-16s, eBioscience), and anti-Ki-67 (clone 16A8, BioLegend). For cytokine detection, cells were stimulated *ex vivo* by incubation for 4–6 h with 50 ng/ml phorbol-12-myristat-13-acetate (PMA), 1 µg/ml ionomycin, and 10 µg/ml Brefeldin A (all obtained from Sigma), fixed and permeabilized as indicated above, and stained with anti-IL-4 (clone 11B11, BD), anti-IL-5 (clone TRFK5, BioLegend), anti-IL-13 (clone eBio13A, eBioscience), anti-IL-17A (clone TC11-18H10.1, BioLegend), and anti-IFNγ (clone xmg1.2, BioLegend), unless otherwise stated. Dead cells were excluded from the analysis using a Zombie viability dye (BioLegend). Fluorescent minus one (FMO) controls were used to properly interpret flow cytometry data, identify, and gate cells, and UltraComp eBeads (eBioscience) were used as single-stain controls to allow for spectral compensation. Data were acquired on the CytoFLEX Platform (Beckman Coulter) and analyzed using FlowJo software (Tree Star Inc.). A MoFlo Astrios EQ cell sorter (Beckman Coulter) was used to purify ILC2. Cells from spleen and MLNs were stained with anti-CD3 (clone 17A1, BioLegend), anti-CD5 (clone 53-7.3, BioLegend), anti-B220 (clone RA3-6B2 eBioscience), anti-NKp46 (clone 29A1.4, BioLegend), anti-CD11b (clone M1/70, eBioscience), anti-CD11c (clone N418, eBioscience), anti-KLRG1 (clone 2F1, BioLegend), anti-ICOS (clone 7E.17G9, BD Bioscience), and anti-ST2 (clone DJ8, MD Biosciences). ILC2 were identified as CD5^–^ B220^–^ CD45R^–^ NKp46^–^ CD11b^–^ CD11c^–^ ICOS^+^ KLRG1^+^ ST2^+/–^ cells. The purity of ILC2 populations was ≥95%, as verified by post-sort flow cytometric analysis.

### Quantitative Real-Time PCR

Samples were lysed with TriFast™ (Peqlab), and RNA was isolated following the instructions of the manufacturer. cDNA was generated using the High-Capacity cDNA Reverse Transcription Kit (Applied Biosystems™) and analyzed using SYBR^®^ Select Master Mix (Thermo Fisher Scientific) on a QuantStudio™ 6 Flex Real-Time PCR Instrument (Thermo Fisher Scientific). Gene expression results were expressed as arbitrary units relative to expression of the housekeeping gene TATA box-binding protein (Tbp), unless indicated otherwise. Primer sequences are as follows: Tbp: 5′-CTACCGTGAATCTTGGCTGTAAAC-3′ and 5′-AATC AACGCAGTTGTCCGTGGC-3′, Btn2a2: 5′-TCAATAACACTCTGCTCAGCCA-3′ and 5′-TCCTTCTCTTCATATTCGGCTTC-3′, H2-Aa: 5′-GGAGGTGAAGACGACATTGAGG-3′ and 5′-CTCAGGAAGCATCCAGACAGTC-3′, H2-Ab1: 5′-GTGTGCAGACACAACTACGAGG-3′ and 5′-CTGTCACTGAGCAGACCAGAGT-3′, Cd80 5′-CCTCAAGTTTCCATGTCCAAGGC-3′ and 5′-GAGGAGAGTTGTAACGGCAAGG-3′, Tnfsf4: 5′-GGAAGAAGACGCTAAGGCTGGT-3′ and 5′-CTGGTAACTGCTCCTCTGAGTC-3′.

### High-Throughput Expression Profiling

Data were obtained from the GitHub repository setup by Björklund et al. [(PMID:26878113), https://github.com/asabjorklund/ILC_scRNAseq/, last accessed on January 28, 2021] and processed as described in the article, with scripts adapted from those provided by the authors on the same GitHub repository. Briefly, RPKM values in./data/ensembl_rpkmvalues_ILC.txt were corrected for batch effects between the tonsil donors using the ComBat() function in the R/Bioconductor sva package [version 3.32.1 (22)]. The 847 genes with higher variation than the spike-in RNAs were then subjected to dimensionality reduction with t-stochastic neighbor embedding (tSNE). For this purpose, the Rtsne() function in the R Rtsne package [version 0.15 (23)] was applied with default parameters except for “initial_dims=10” and “theta=0.001.” For identification of markers of BTN2A2-expressing cells and single-cell heat map, we used Seurat [version 4.0.3 (24)]. Gene ontology analysis was performed using DAVID [version 6.8 (25)]. Bulk RNA-Seq was derived from Shih et al. [(PMID: 27156451), GSE77695] and processed as described in the original article. Regularized log transformation, base means, and z scores were calculated in R (version 4.1.0).

### Histology

Small intestine tissue was fixed in 4% formaldehyde (Roth) for 4 h at room temperature and embedded in paraffin. Slices of 4-µm thickness were cut on a microtome before staining with periodic acid–Schiff reagent, or hematoxylin and eosin. Pictures were acquired on a BZ-X710 fluorescence microscope (Keyence).

### Statistical Analysis

Results represent mean ± standard error of the mean (SEM). Statistical analyses were performed using Student’s t-test for single comparison, or analysis of variance (ANOVA) test for multiple comparisons (one-way or two-way ANOVA followed by Tukey’s or Bonferroni’s multiple-comparison test, respectively), unless otherwise specified. Experiments were conducted at least three times, unless otherwise stated in the figure legends. Graph generation and statistical analyses were performed using the Prism version 8 software (GraphPad, La Jolla, CA).

## Results

### Btn2a2^-/-^ Mice Show Stronger Type-2 Immune Responses and Reduced Worm Burden

**Figure 1:**
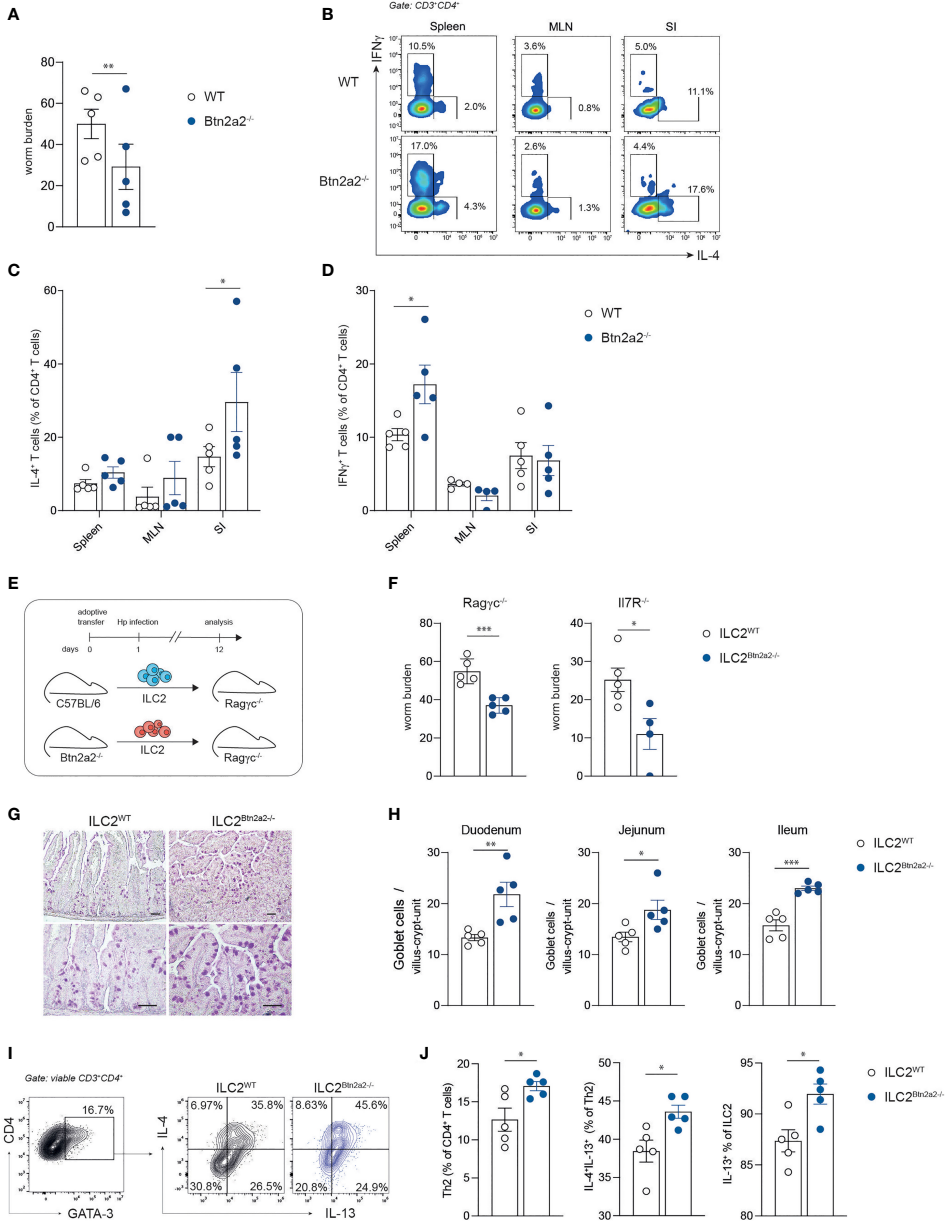
Btn2a2^-/-^ ILC2 drive elevated type 2 responses against helminth infections. **(A)** Sex- and age-matched WT and Btn2a2^-/-^ mice were infected with Hp, and the number of adult worms in the intestines of infected animals was quantified on day 37 after infection. **(B)** Representative FACS plots of CD4^+^ T cells isolated from spleen, MLN, and small intestinal lamina propria of infected animals 37 dpi and stained for IL-4 and IFNγ cytokine production and corresponding dotplots of IL-4^+^ T cells **(C)** and IFNγ^+^ T cells **(D)**. **(E)** Schematic overview of experimental plans. Sorted WT or Btn2a2^-/-^ ILC2 were adoptively co-transferred with CD4^+^ T cells into Ragγc^-/-^ and Il7R^-/-^ mice and subsequently infected with 200 L3 stage larvae of Hp. **(F)** Mice were sacrificed on days 10 and 12 postinfection, respectively, and adult worm counts in small intestinal tissues were determined. **(G)** AB-PAS staining was performed from small intestine paraffin sections of Ragγc^-/-^ mice. Scale bars indicate 50 µm. Pictures show results of one representative animal. **(H)** Goblet cells per villus-crypt-unit were enumerated. **(I, J)** Lamina propria lymphocytes of Ragγc^-/-^ mice were isolated and re-stimulated with PMA/ionomycin in the presence of Brefeldin A for 4 h before intracellular cytokine staining. Significance was assessed using unpaired Student’s t test **(A, F, H, J)** or two-way ANOVA **(C, D)**. Data are representative of three **(A–D)** and two **(E–I)** independent experiments. Data are shown as means ± SEM. *p < 0.05; **p < 0.01; ***p < 0.001.

To address the relevance of Btn2a2 on type-2 immune responses in general, we infected wild-type mice (WT) and mice deficient for Btn2a2 (Btn2a2^-/-^) with the natural, murine parasitic helminth *Heligmosomoides polygyrus bakeri* (Hp). Btn2a2^-/-^ mice showed significantly lower adult worm burden in the intestine (**Figure 1A**). The decreased worm burden in Btn2a2^-/-^ mice may be the result of an amplified local type-2 immune response at the site of infection, shown by increased frequencies of IL-4^+^ Th2 cells in the small intestine of Hp-infected Btn2a2^-/-^ mice (**Figures 1B, C**). No difference in IL-4^+^ Th2 frequency was observed in spleen and mesenteric lymph nodes (MLN) (**Figures 1B, C**). Interestingly, the frequency of IFNγ^+^ cells was significantly elevated in the spleen of Hp-infected Btn2a2^-/-^ mice (**Figures 1B, D**). As IFNγ counteracts IL-4 responses, the net effect of the absence of Btn2a2 may be dampened (26). These data suggest that absence of Btn2a2 expression provokes a stronger systemic T cell response, whereby locally increased type-2 immune responses promote effective Hp expulsion. However, these data are derived from complete Btn2a2^-/-^ mice, not allowing conclusions on the responsible Btn2a2 expressing cell type.

### Specific Loss of Btn2a2 Expression on ILC2 Mirrors the Immune Phenotype of Btn2a2^-/-^ Mice

ILC2 contribute to the expulsion of parasitic helminths and induce strong type-2 immune responses (7). Type-2 immune responses are initiated by ILC2 cells releasing type 2 cytokines and thereby inducing Th2 cells, a necessity for effective helminth expulsion (7). Therefore, we speculated that the loss of Btn2a2 specifically on ILC2 is in part responsible for the observed phenotype in Btn2a2^-/-^ mice. To address this hypothesis, we adoptively co-transferred purified ILC2 from IL-25/IL-33 minicircle-treated (mc) WT and Btn2a2^-/-^ mice together with naïve WT T cells into ILC-deficient Ragγc^-/-^ or IL-7R^-/-^ mice (**Figure 1E**). Ragγc^-/-^ in addition lack B and T cells while IL-7R^-/-^ mice show reduced numbers of T cells (27, 28). Interestingly, both Hp-infected Ragγc^-/-^ and IL-7R^-/-^ mice receiving ILC2^Btn2a2-/-^ displayed lower worm burden compared to the respective mice receiving ILC2^WT^ (**Figure 1F**), reflecting the results obtained in full Btn2a2^-/-^ mice (**Figure 1A**). Moreover, histological analysis of the small intestine revealed increased goblet cell numbers per villus in the duodenum, jejunum, and ileum of ILC2^Btn2a2-/-^recipient Ragγc^-/-^ mice (**Figures 1G, H**). To address the question if the lower worm burden in the ILC2^Btn2a2-/-^recipient Ragγc^-/-^ mice is due to an elevated type-2 response, we analyzed ILC2 and Th2 cells after the adoptive transfer. Ragγc^-/-^ mice reconstituted with ILC2^Btn2a2-/-^ showed higher frequencies of Th2 cells and IL-4^+^/IL-13^+^ Th2 cells as well as higher frequency of IL-13^+^ ILC2 (**Figures 1I, J**), while the frequencies of ILC2 was unchanged (**Supplementary Figure 1D**). These data indicate that loss of Btn2a2 on ILC2 negatively regulates type-2 immune responses to Hp infection.

### Expression of Btn2a2 Is Upregulated in Stimulated ILC2

**Figure 2:**
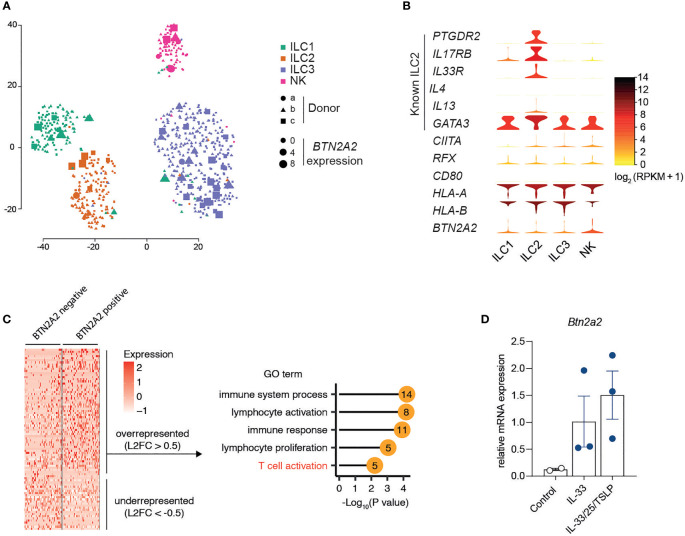
Expression of Btn2a2 is upregulated in stimulated ILC2. **(A)** t-SNE graph of single-cell RNA-sequencing data from 648 sort-purified human ILCs reveals heterogeneous Btn2a2 expression throughout clusters. Each symbol represents an individual cell. Colors indicate marker phenotype, and symbols, donor origin. **(B)** Expression distribution (violin plots) in each population of human ILC (horizontal axis) for selected genes. Colors indicate mean expression (key). **(C)** Heatmap reports scaled expression of discriminate gene sets for BTN2A2 expressing cells (BTN2A2 > 0). Discriminative genes for BTN2A2 expressing cells that were overrepresented (Log2FC ≥ 0.5) were used for gene ontology (GO) analysis using DAVID and selected GO terms, and their p-values are represented as lollipop graph where numbers indicate the number of genes that fall into the respective GO term. **(D)** qPCR for Btn2a2 of sorted ILC2 after *in vitro* stimulation with the indicated cytokines (50 ng/ml). Data for ILC scRNA-seq came from ref. 29.

To assess BTN2A2 expression in ILC, we analyzed publicly available human single-cell RNA-sequencing data of steady-state ILC (29). Interestingly, BTN2A2 was expressed in specific subsets of ILC comprising ~8% of total cells and clustered together with the four major ILC (**Figure 2A**), suggesting that BTN2A2 expression is rather associated with a functional state or cellular subpopulation as opposed to being ILC2-specific. The frequency of BTN2A2-expressing ILC2 was similar to that of IL-13, an important effector molecule of ILC2 (**Figure 2B**). Consistent with reports of murine ILC2 (7), human ILC2 also expressed MHCII genes, such as HLA-A and HLA-B (**Figure 2B**), highlighting their potential to prime T cells. Moreover, we also found a subset of ILC2-expressing CIITA and RFX, which we have previously shown to regulate BTN2A2 gene expression together with MHCII genes (18). Seurat (24) analysis revealed 88 genes that were significantly (p ≤ 0.05) over- or underrepresented in BTN2A2-expressing cells (**Figure 2C** and **Supplementary Table 1**). For a functional analysis, we restricted the overrepresented genes to a log2-fold change of ≥0.5 and performed gene ontology (GO) using DAVID. Strikingly, we found these genes to fall into GO terms specifically related to T cell activation (**Figure 2C**) supporting the observation that BTN2A2 expression correlates with the expression of genes that are implicated in T cell activation (**Figure 2C**). Based on the observation that BTN2A2 was expressed on a subset of ILC2 in steady state and revealed a similar expression pattern as IL-13, we hypothesized that it is expressed by activated ILC2. To test this hypothesis, we sort-purified ILC2 from WT and Btn2a2^-/-^ mice and stimulated them with IL-33 or IL-33, IL-25 and TSLP. Btn2a2 RNA expression increased after stimulation with IL-33 and was further upregulated following combined treatment with IL-25, IL-33, and TSLP (**Figures 2C, D**), the three main cytokines relevant for helminth expulsion (30). We also analyzed RNA-Seq of murine ILC (31); however, we were unable to find expression patterns but could confirm Btn2a2 expression in ILC2 upregulated after IL-25/IL-33 stimulation (**Supplementary Figure 2**). The ligand binding to Btn2a2 expressed on the cell surface, and the cell types expressing this ligand are so far unknown (18). The upregulation of Btn2a2 on ILC2 after stimulation with IL-25/IL-33/TSLP points to a role of Btn2a2 signaling events responsible for the enhanced ILC2^Btn2a2-/-^mediated type-2 immune responses in helminth-infected mice.

### ILC2 Numbers and ILC2 Effector Molecules in Steady State Are Unchanged in Btn2a2^-/-^ Mice

**Figure 3:**
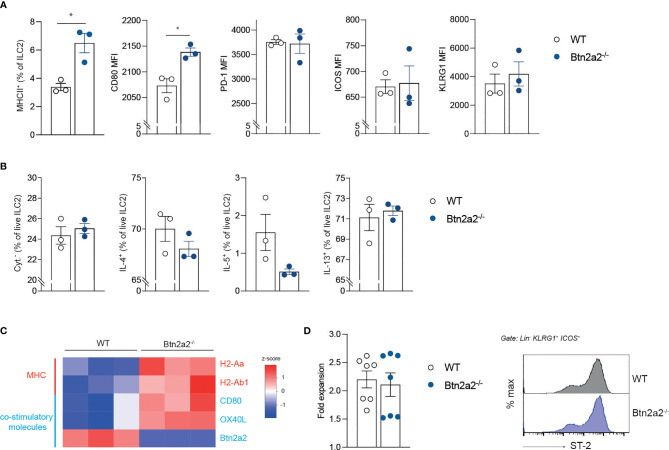
Btn2a2 does not mediate expression of effector molecules on ILC2 in steady state. **(A)** Flow cytometric analysis of surface molecules of ILC2 of WT and Btn2a2^-/-^ mice isolated from spleens. **(B)** Sorted-purified ILC2 were expanded *in vitro* for 5 days [IL-2, IL-7, IL-25, IL-33 (each 50 ng/ml) and TSLP (10 ng/ml)], stimulated for the final 4 h with PMA/ionomycin in the presence of monensin and stained intracellularly for IL-4, IL-5, and IL-13. **(C)** Heatmap showing heatmap.2 calculated z-scores of genes analyzed by qPCR after *in vitro* stimulation with IL-25/IL-33 (50 ng/ml) from WT and Btn2a2^-/-^ ILC2. **(D)** Expansion of ILC2 after 3 days of *in vitro* expansion was enumerated, and ST-2 expression was analyzed. Data are representative of two independent experiments. Data are shown as means ± SEM. *p < 0.05.

To exclude that the phenotype of Btn2a2^-/-^ mice is due to a defect in ILC2 development, we analyzed ILC2 numbers in steady state in WT and Btn2a2^-/-^ mice from spleen, MLNs, small intestine (SI), and lung. ILC2 from naïve WT vs. Btn2a2^-/-^ mice (gating see **Supplementary Figures 3A, B**) did not show any differences in numbers in spleen, MLN, SI, and lung (**Supplementary Figure 3C**). The mechanisms responsible for ILC2-mediated immune regulation include the expression of cell surface effector molecules or secretion of cytokines (32). To address the mechanism underlying the ILC2^Btn2a2-/-^driven upregulation of type-2 immune responses observed in helminth infection, we analyzed common effector surface molecules in ILC2 and their capacity to produce type-2 effector cytokines. ILC2 isolated from naïve Btn2a2^-/-^ and littermate control mice did not differ in their expression of common effector molecules in lung and SI (**Supplementary Figures 3D–K**). Lung and SI ILC2 isolated from Btn2a2^-/-^ and littermate control mice did not differ in their capacity to produce the known type-2 effector cytokines IL-4, IL-5, and IL-13 (**Supplementary Figures 4B–D**), and also the levels of IL-4, IL-5, and IL-13 were unchanged in purified, *in vitro* expanded splenic ILC2^Btn2a2-/-^ cells following ILC2 expansion cytokine cocktail stimulation (**Figure 3B**).

Flow cytometry analysis revealed an upregulation of MHCII on ILC2^Btn2a2-/-^ compared to ILC2^WT^ in spleen and MLN (**Figure 3A**), whereas there was no upregulation of MHCII in ILC2 isolated from lung and SI from naïve mice (**Supplementary Figure 3K**). CD80 expression on splenic ILC2^Btn2a2-/-^ was also elevated, but remained unchanged on ILC2 from lung and SI (**Supplementary Figure 3J**). KLRG1, PD-1, OX40L, or ICOS expression on ILC2^Btn2a2-/-^ cells compared to ILC2^WT^ remained unchanged in spleen, SI, and lung (**Figure 3A** and **Supplementary Figures 3E–I**). Analysis of the expression of ILC2 effector genes after IL-25/IL-33 stimulation revealed upregulation of co-stimulatory molecules OX40L and CD80 as well as MHCII genes H2-Ab1 and H2-Aa in mice deficient for Btn2a2 (**Figure 3C**). This suggests a possible involvement of Btn2a2 on the identified co-stimulatory potential of ILC2 (7, 8, 33). To exclude a general proliferation defect of ILC2, comparisons of purified splenic ILC2 expansion rates and also proliferation from PMA/ionomycin-activated ILC2 from lung and SI from WT vs. Btn2a2^-/-^ mice did not show any differences (**Figure 3D** and **Supplementary Figure 4A**), nor did we observe different activation states, as shown by ST-2 staining of ILC2 from spleen, lung, and SI (**Figure 3D** and **Supplementary Figure 3G**).

### Btn2a2 on ILC2 Supresses Type-2 Immune Responses *In Vitro*

**Figure 4:**
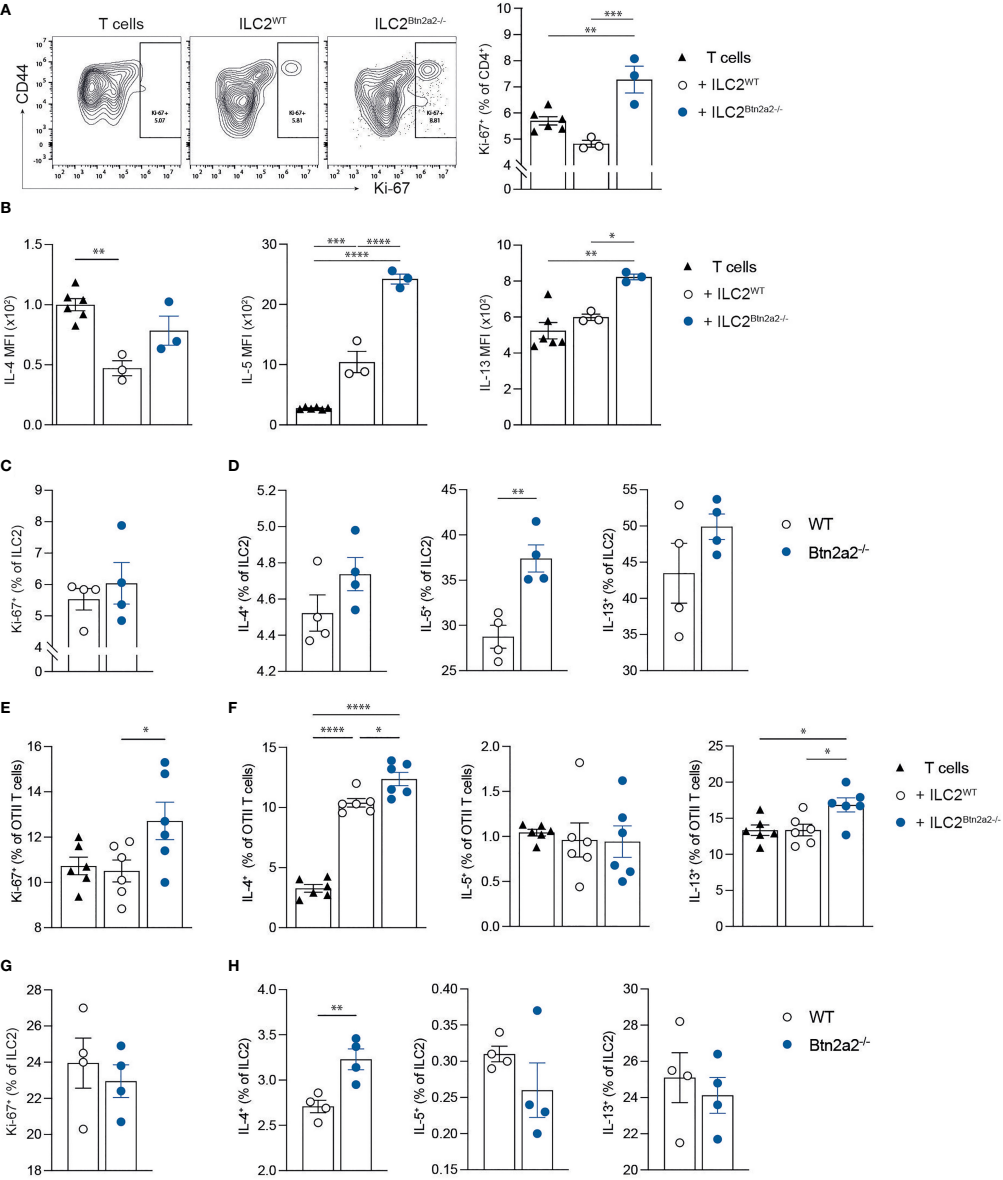
Btn2a2 negatively regulates the development of an ILC2-induced type 2 response *in vitro*. **(A–D)** CD4^+^ T cells were cultured under activating conditions (1:1 Dynabeads) alone or with Btn2a2^-/-^ ILC2 or WT ILC2 and proliferation T cells **(A)** was analyzed by flow cytometry. **(B)** Supernatants from cocultured T cells were analyzed for Th2 cytokines utilizing LEGENDplex. **(C, D)** Proliferation **(C)** and cytokine production **(D)** of ILC2 was analyzed by flow cytometry. **(E–H)** CD4^+^ OTII T cells were cocultured with Btn2a2^-/-^ ILC2 or WT ILC2 for 96 h in the presence of OVA peptide. OTII T cell proliferation **(E)** and cytokine production **(F)** was analyzed by flow cytometry. ILC2 proliferation **(G)** and cytokine production **(H)** were analyzed by flow cytometry. Data are representative of three independent experiments. Data are shown as means ± SEM. *p < 0.05; **p < 0.01; ***p < 0.001; ****p < 0.0001.

In order to further expand on the observation that ILC2^Btn2a2-/-^ cells initiate stronger type-2 immune responses, we cocultured ILC2^Btn2a2-/-^ or ILC2^WT^ cells with anti-CD3/CD28-activated WT CD4^+^ T cells to analyze their proliferation and cytokine release. Cocultures with ILC2^Btn2a2-/-^ showed significantly increased proliferation of CD4^+^ T cells compared to cocultures with ILC2^WT^ (**Figure 4A**). Coculture of T cells with ILC2^Btn2a2-/-^ caused a significantly increased production of IL-5 and IL-13 but not of IL-4 measured in the coculture supernatants compared to T cells cocultured with ILC2^WT^ cells (**Figure 4B**). Analyzing the ILC2 from ILC2-T cell cocultures showed no increase in proliferation (**Figure 4C**) and no differences in IL-4 and IL-13 expression but significantly elevated expression of IL-5 in ILC2^Btn2a2-/-^ (**Figure 4D**).

Since we observed an increased expression of the co-stimulatory molecules CD80 and MHCII on ILC2^Btn2a2-/-^ (**Figures 3A, C**), we further analyzed if the type-2 promoting effects initiated by ILC2^Btn2a2-/-^ is antigen-specific. Therefore, ILC2^Btn2a2-/-^ or ILC2^WT^ cells were cocultured with OVA-specific CD4^+^ cells from OTII TCR transgenic mice in the presence of ovalbumin peptide (OVA_323-339_). OTII CD4^+^ T cells cocultured with ILC2^Btn2a2-/-^ showed increased proliferation compared to OTII CD4^+^ T cells cultured with ILC2^WT^ cells (**Figure 4E**). Analysis of cytokine production of OTII CD4^+^ T cells showed that coculture with ILC2^Btn2a2-/-^ resulted in significantly elevated frequencies of IL-4 and IL-13, but not of IL-5 (**Figure 4F**). This effect could be attributed to antigen-dependent interactions between OTII T cells and ILC2, as cocultures without OVA_323-339_ resulted in significantly reduced OTII T cell proliferation and cytokine production (**Supplementary Figures 5A, B**). Moreover, in the absence of OVA_323-339_ the differences of OTII T cell proliferation and cytokine production were evened out, suggesting that Btn2a2 is directly involved into antigen-dependent T cell priming (**Supplementary Figures 5A, B**). Analyzing the ILC2 from ILC2-T cell cocultures showed no increase in proliferation (**Figure 4G**) and no differences in IL-5 and IL-13 expression but elevated expression of IL-4 in ILC2^Btn2a2-/-^ (**Figure 4H**). To further exclude a general defect of T cells in Btn2a2^-/-^ mice, we analyzed CD4^+^ T cells from naïve Btn2a2^-/-^ and littermate control mice from lung and SI in terms of their identity and capacity to produce type-2 and type-1 effector cytokines as well as Foxp3 expression and proliferation. We did not observe any differences between naïve Btn2a2^-/-^ and littermate control mice in type-2, type-1, and type-3 effector cytokines IL-4, IL-5, IL-13, IFNγ, and IL-17 respectively as well as Foxp3 expression and proliferation (**Supplementary Figures 6A–C, E**). Moreover, frequencies of Tbet+, GATA3+, and RORγt+ T cells from SI and lung were unchanged (**Supplementary Figures 6D, F**).

### Btn2a2 on ILC2 Suppresses Antigen-Specific Type-2 Immune Responses *In Vivo*

**Figure 5:**
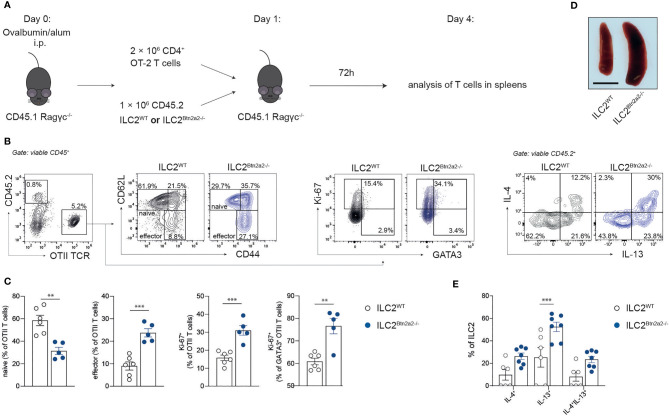
Btn2a2 negatively regulates the development of an ILC2-induced type 2 response *in vivo*. **(A)** Schematic of experimental plans. Ragγc^-/-^ mice were immunized with alum-precipitated OVA, and after 24 h CD4^+^ OTII T cells were adoptively co-transferred together with Btn2a2^-/-^ ILC2 or WT ILC2. 72 h post transfer, spleens were analyzed for OTII T cell and ILC2 response by flow cytometry. **(B)** Representative photograph of spleens of Ragγc^-/-^ mice. **(C)** Gating strategy and representative flow cytometry graphs of OTII T cell Btn2a2^-/-^ (blue) and WT (black) ILC2 transferred Ragγc^-/-^ mice. **(D)** Frequencies of naïve and effector OTII T cells recovered from spleens and quantification of their proliferation. **(E)** Analysis of ILC2 cytokine expression from Btn2a2^-/-^ ILC2 and WT ILC2 recovered from spleens of Ragγc^-/-^ recipients 72 h post transfer. Data are representative of three independent experiments. Data are shown as means ± SEM. **p < 0.01; ***p < 0.001.

Next, to examine if Btn2a2 expression on ILC2 generally promotes immune suppression by ILC2 in an antigen-specific manner *in vivo*, we adoptively transferred ILC2^Btn2a2-/-^ or ILC2^WT^ cells together with OTII CD4^+^ T cells into Ragγc^-/-^ mice pre-immunized with OVA (**Figure 5A**). Upon recovery of total T cells, naïve T cells, T effector cells (T_eff_), Th2 cells, and ILC2 in Ragγc^-/-^ mice (**Figure 5B**), we observed that in mice receiving ILC2^Btn2a2-/-^, the frequency of T_eff_ was significantly increased compared to mice receiving ILC2^WT^ whereas frequencies of naïve T cells were significantly decreased (**Figure 5C**). Proliferation of CD4^+^ T cells as well as of CD4^+^ Th2 cells was also significantly increased in mice receiving ILC2^Btn2a2-/-^ compared to mice receiving ILC2^WT^ (**Figure 5C**). This increase in proliferation was mirrored in an increase in spleen size (**Figure 5D**). Moreover, IL-13 cytokine production from ILC2 was increased in mice receiving ILC2^Btn2a2-/-^ compared to mice receiving ILC2^WT^ (**Figure 5E**). These data indicate that ILC2-specific Btn2a2 is able to suppress CD4^+^ effector T cell responses independent of helminth infections in an antigen-specific manner. When analyzing the ILC2 that were recovered after adoptive transfer, we observed that the frequencies of IL-13^+^ ILC2 were significantly increased in mice receiving ILC2^Btn2a2-/-^ while IL-4^+^ and IL-4/13^+^ ILC2 were elevated but without reaching significance (**Figure 5E**). Notably, IL-13 response by ILC2 was diminished when transferred without OTII T cells (**Supplementary Figure 7B**), highlighting the interdependency of ILC2 and CD4^+^ T cell in responding to helminth infections (7, 34).

## Discussion

Our findings indicate a critical role of the co-stimulatory molecule Btn2a2 in the regulation of the ILC2-T cell cross talk during inflammatory type 2 responses. ILC2 and Th2 cells interact on multiple levels, ILC2 regulate CD4^+^ T cell responses and in turn receive feedback from these cells during the ILC2 T cell cross talk. In mice lacking ILC2, induction of type 2 responses upon helminth infection and after challenge with house dust mite antigen and papain is dramatically reduced (5–7), indicating a substantial role for ILC2 in induction of Th2 cell responses. Impaired IL-4 and/or IL-13 signaling results in reduced type 2 responses (35–37), and IL-4 is secreted by cells of the innate immune system such as basophils and mast cells but also by ILC2 (9, 38–40). Activated CD4^+^ T cells in turn can induce ILC2 proliferation, and this results in an upregulation of IL-4 mRNA (9) and IL-5/IL-13 secretion in ILC2 (8), suggesting that Th2 cells can induce type 2 cytokines in ILC2. It has been shown that ILC2 are important for the mounting of a proper type 2 T cell response during helminth infections (41), for allergy models (5, 7), and in the context of inflammatory arthritis (3). Using Btn2a2^-/-^ mice, Btn2a2 has been shown to act as a co-inhibitory molecule that restrains T cell-mediated immunity (18). However, the responsible Btn2a2-expressing cell type remained elusive. In the present study, we could show that mice deficient for Btn2a2 specifically on ILC2 displayed elevated levels of effector IL-4/IL-13^+^ T cells resulting in better clearance of helminths, suggesting that Btn2a2 on ILC2 is responsible for limiting the local T cell response upon Hp infection. T cell responses against intestinal nematodes are enhanced in the presence of ILC2 (7, 34), and it could be of interest to analyze the response of T cell-deficient mice infected with Hp receiving Btn2a2^-/-^ T cells alone without ILC2. However, since in our analysis of the global Btn2a2^-/-^ mouse in steady state we did not observe any proliferation or polarization defects of T cells or differences in their capacity to produce cytokine, we assume that the shown superior clearance of the pathogen in Btn2a2^-/-^ mice is a result of ILC2-T cell crosstalk and not of a defect in T cells per se. However, it has to be pointed out that this cannot be said with certainty without a T cell only transfer control. In addition, although our in vitro data strongly suggest a role of Btn2a2 in T cell: ILC2 crosstalk, whether Btn2a2^-/-^ ILC2 could elicit a greater response without T cells remains to be investigated.

ILC2 were recently shown to express MHCII as well as co-stimulatory molecules including B80/86, ICOS, and OX40L, and these were upregulated in the context of type 2 infection and inflammation (42). Therefore, not only do regulation and fine tuning of type 2 immune responses depend on cytokine secretion but also co-stimulatory signals and antigen presentation are involved in the induction of an effective Th2 response to helminth infections. Inhibiting T cell co-stimulation by blocking CD80 and CD86 signaling reduced IL-4 expression and Th2 expansion in response to Hp (43, 44); however, blocking of CD80 or CD86 alone had little effect (43). Similarly, absence of the CD80/CD86 receptor, CD28, had no impact in early Th2 response (43), suggesting that other mechanisms for Th2 co-stimulation during Hp infection exist. For example, another co-stimulatory molecule, OX40L, has been shown to be required to promote IL-4 production from T cells without affecting Th2 cell expansion (45).

Analysis of open access RNA-Seq data (46) revealed that ILC2 expressed Btn2a2 and that the expression is upregulated upon activation together with other co-stimulatory molecules such as CD80/86 and MHCII. We could confirm Btn2a2 expression from these open-access-derived data in purified ILC2 after stimulation with IL-33/25/TSLP. Of note, Btn2a2 mRNA and protein levels in steady state were barely detectable but increased during inflammatory responses, pointing to a negative feedback loop in suppressing already initiated inflammatory responses. Upon infection, ILC2 rapidly expand and exert their function in promoting type 2 immune response by means of effector molecule expression such as PD-1 (47–50) and by secretion of type 2 cytokines IL-4, IL-5, and IL-13 (51, 52). We did not observe a defect in expansion of ILC2^Btn2a2-/-^, or in effector molecule surface expression, or in cytokine release after stimulation with IL-25/IL-33/TSLP, the most important factors that induce ILC2 activation (51, 52). Also, when analyzing ILC2 from the lungs and small intestine of naïve Btn2a2^-/-^ and control littermate mice, there was no difference in effector molecule surface expression and cytokine production. However, after stimulation with IL-25/IL-33/TSLP, Btn2a2-deficient ILC2s expressed elevated levels of co-stimulatory molecules CD80 and OX40L as well as MHCII. MHCII upregulation on ILC2 may interact with the TCR on CD4^+^ T cells, as well as CD80/86 with CD28 and OX40L with OX40, to induce further production of IL-4/IL-5/IL-13 and GATA3, and this upregulation may lead to CD4^+^ Th2 cell differentiation. This was underlined by our finding that in cocultures with naïve T cells, only Btn2a2-deficient ILC2 displayed elevated levels of effector cytokines IL-4/IL-5/IL-13 to promote type 2 T cell responses. It was already shown that upon stimulation, ILC2 activate T cells and skew the response toward a type 2 effector T cell response (51, 52). The activated Th2 cells in turn further stimulate ILC2 to produce effector cytokines, a process called the ILC2-T cell cross talk (2, 7, 53, 54).

Interestingly, analysis of the open-access RNA-Seq data (46) also revealed that Btn2a2 is expressed as well in ILC1, ILC3, and NK cells in addition to ILC2. For ILC3 and NK cells, it has been shown that in addition to the secretion of cytokines, immunoreceptor ligation can stimulate T cell responses in an antigen-dependent manner and thereby equipping these cells with adaptive features (33, 55), as it was shown for ILC2. One may speculate that Btn2a2 may therefore play a similar role in these ILC subsets as we showed here in our study, and this expression in other antigen-presenting ILC subsets further strengthens our hypothesis of Btn2a2 as a co-stimulatory molecule on ILC. Moreover, several studies show that, similar to T helper cells, the different ILC subsets can modify their phenotype and function based on environmental cues, a phenomenon named “plasticity” (56). Recent advances in the ILC2 field have led to the discovery that ILC2s can promptly shift to functional IFN-γ-producing ILC1s or IL-17-producing ILC3s, depending on the cytokines and chemokines produced by antigen-presenting cells or epithelial cells (56–58). A role of Btn2a2 in altering the T cell responses per se can be excluded since analysis of T cells from steady-state lungs and small intestines from Btn2a2^-/-^ mice did not differ in their proliferative capacity, production of cytokines, and polarization, thereby further promoting a role of Btn2a2 as co-stimulatory molecule important in ILC-T cell cross talk.

Primary infections with helminth induced IL-3, IL-4, IL-5, and IL-9 gene expression in the intestinal site (mesenteric lymph nodes and Peyer’s patches), leading to high IL-4, IL-5, IL-9, IL-10, and IL-13 protein concentrations in mesenteric lymph node, spleen, and lamina propria mononuclear cells cultured with parasite antigen (59–61). Interestingly, in an infection model with *N. brasiliensis*, the expression of MHCII was found to be elevated in LN-, spleen-, and Peyer’s Patch (PP)-derived ILC2s than on peritoneal lavage-, bronchoalveolar lavage-, and lung-derived ILC2s (7). Therefore, factors released at the site of the first response to infection may also affect MHCII expression and thereby further antigen-dependent steps.

Blocking T cell co-stimulation by inhibiting signaling through both CD80 and CD86 on ILC2 resulted in reduced IL-4 expression in response to Hp; however, no impact was observed on the innate IL-5 response (43, 44). In line with this finding, we observed that significant elevation in IL-4 production from Btn2a2^-/-^ ILC2 was found to be antigen-dependent, while impact on IL-5 production from Btn2a2^-/-^ ILC2 appeared to be antigen-independent. Moreover, ILC2-derived IL-4 drives Th2 differentiation during Hp infection (34), and the elevated production of IL-4 by Btn2a2^-/-^ ILC2 and OTII cells indicates that the negative impact of Btn2a2 on ILC2 on the expression of IL-4 is antigen-dependent. In mice where ILC2 are genetically ablated, IL-5 production by CD4+ T cells drops in MLN during helminth infection resulting in ablated expulsion of worm (7). IL-5 production by CD4+ T cells was not affected in antigen-dependent cocultures with ILC2^Btn2a2-/-^. Since we did not observe a defect of ILC2^Btn2a2-/-^ in steady state or after stimulation beside an upregulation of co-stimulatory molecules, we speculated that Btn2a2 plays a role in this ILC2-T cell cross talk. *In vitro* cocultures of ILC2 with T cells showed increased T cell proliferation if Btn2a2 was missing and decreased T cell proliferation after sBtn2a2 supplementation. This finding supports the role of Btn2a2 as being a negative costimulatory molecule in the ILC2-T cell cross talk. We observed an increase in Th2 cytokine cocultures with Btn2a2-deficient ILC2s, as compared to WT ILC2s–T-cell cocultures (**Figure 5B**). In contrast, IL-5 was not elevated in antigen-dependent cocultures, where OTII T cells were used. Interestingly, there have been similar findings in studies where B80/86-blocking antibodies or deficient mice were used. Since MHCII was upregulated on ILC2^Btn2a2-/-^, we further analyzed antigen-specific effects in the ILC2-T cell cross talk. Therefore, *in vivo* adoptive transfer of WT or Btn2a2-deficient ILC2 together with OTII T cells into OVA-immunized Ragγc^-/-^ resulted in increased frequencies of OTII T cells and IL-13^+^ ILC2. Hence, Btn2a2 acts as effective T cell-inhibiting molecule on ILC2, which is induced by infection or during inflammation. We show that Btn2a2^-/-^ ILC2 have increased levels of MHCII and co-stimulatory molecules, which is likely a consequence of ILC2-T-cell-interactions *in vivo*. 

We have previously shown that Btn2a2 is co-regulated with the MHC-II machinery (18) in a CIITA-dependent manner. Additionally, another study could observe reduced T cell receptor signaling in the presence of Btn2a2-Fc *in vitro*, as shown by reduced Zap70, CD3ϵ, and Erk phosphorylation (16). Thus, it is likely that Btn2a2 acts at the epicenter of T cell priming. ILC2 were recently shown to express MHCII as well as co-stimulatory molecules including B80/86, ICOS, and OX40L, and these were upregulated in the context of type 2 infection and inflammation (42). While we believe that ILC2 can attenuate the T cell response by expressing Btn2a2 in the context of MHCII, we also appreciate the possibility that, *in vivo*, ILC2 can exert this action as a bystander APC, while professional APCs display the antigen. Moreover, it was shown that Ag-dependent interactions between ILC2 and T cells result in mutually activating cross talk. We show that Btn2a2^-/-^ ILC2 have increased levels of MHCII and co-stimulatory molecules, which is likely a consequence of ILC2–T-cell interactions *in vivo*.

Taken together, our data highlight Btn2a2 as prominent immunomodulatory molecule on ILC2 under inflammatory conditions that modulates the cross talk between ILC2s and T cells.

## Data Availability Statement

The datasets presented in this study can be found in online repositories. The names of the repository/repositories and accession number(s) can be found in the article/**Supplementary Material**.

## Ethics Statement

The animal study was reviewed and approved by Regierung von Unterfranken.

## Author Contributions

MF, MZ, and KS contributed to the conception and design of the study. MF, YO, and AS conducted the experiments and acquired and analyzed the data. MF and LT performed the statistical analysis. SW and LT wrote sections of the manuscript. MF wrote the first draft of the manuscript. GS, MZ, and KS designed the research studies and wrote the manuscript. All authors contributed to the manuscript revision and read and approved the submitted version.

## Funding

This study was very kindly funded by Dr. Rolf Schwiete Stiftung and the Else Kröner-Fresenius Foundation. Additional funding was received by the Deutsche Forschungsgemeinschaft (DFG, German Research Foundation) DFG-SPP1937 start-up grant and the Interdisciplinary Centre for Clinical Research, Erlangen (IZKF).

## Conflict of Interest

The authors declare that the research was conducted in the absence of any commercial or financial relationships that could be construed as a potential conflict of interest.

## Publisher’s Note

All claims expressed in this article are solely those of the authors and do not necessarily represent those of their affiliated organizations, or those of the publisher, the editors and the reviewers. Any product that may be evaluated in this article, or claim that may be made by its manufacturer, is not guaranteed or endorsed by the publisher.
