# Supplementary material for: Btn2a2 Regulates ILC2–T Cell Cross Talk in Type 2 Immune Responses
Source: Front Immunol. 2022 Jan 25;13:757436. doi: 10.3389/fimmu.2022.757436 (PMC8821520; doi:10.3389/fimmu.2022.757436)

Supplementary Material

# Supplementary Figures and Tables


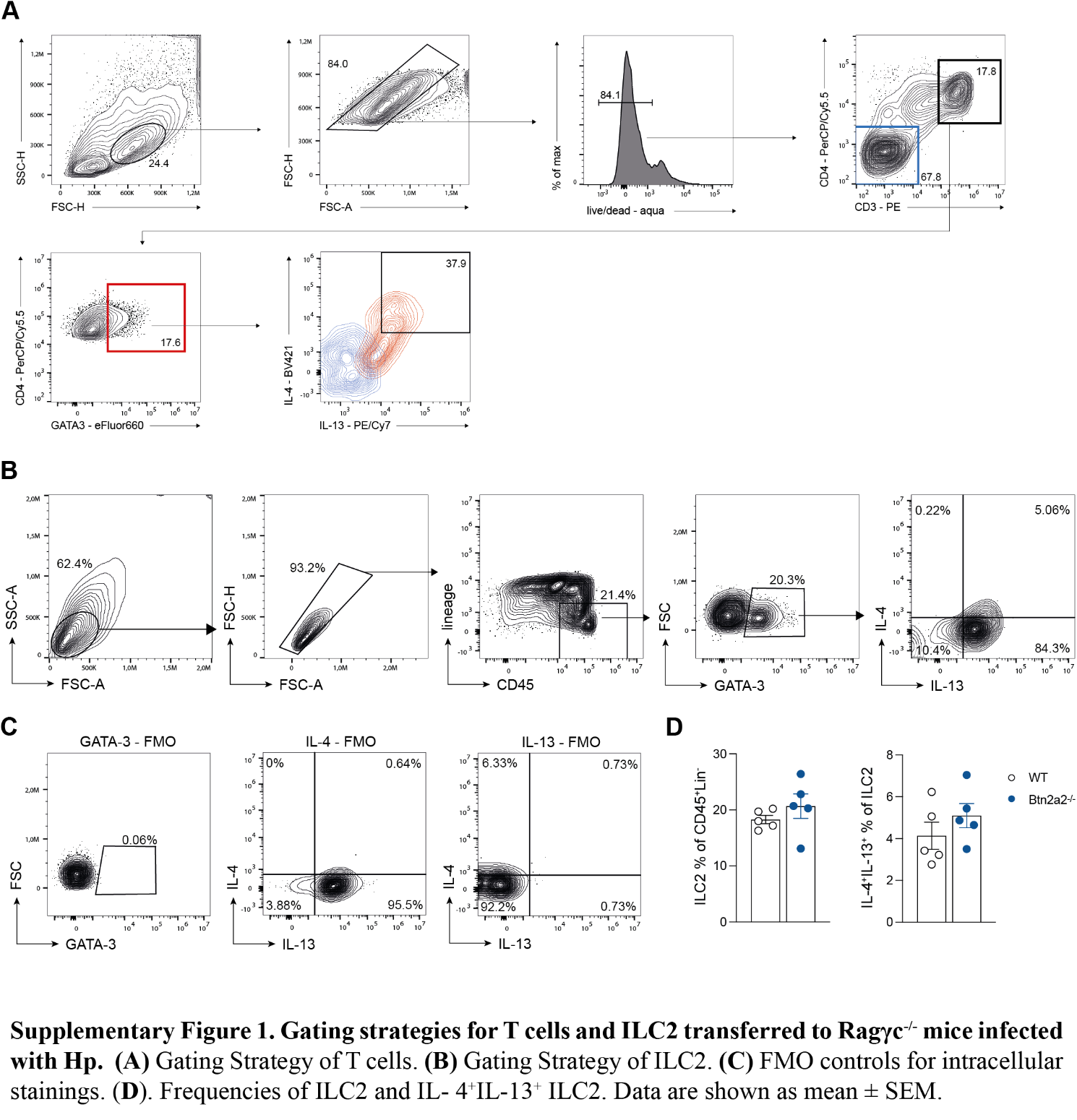


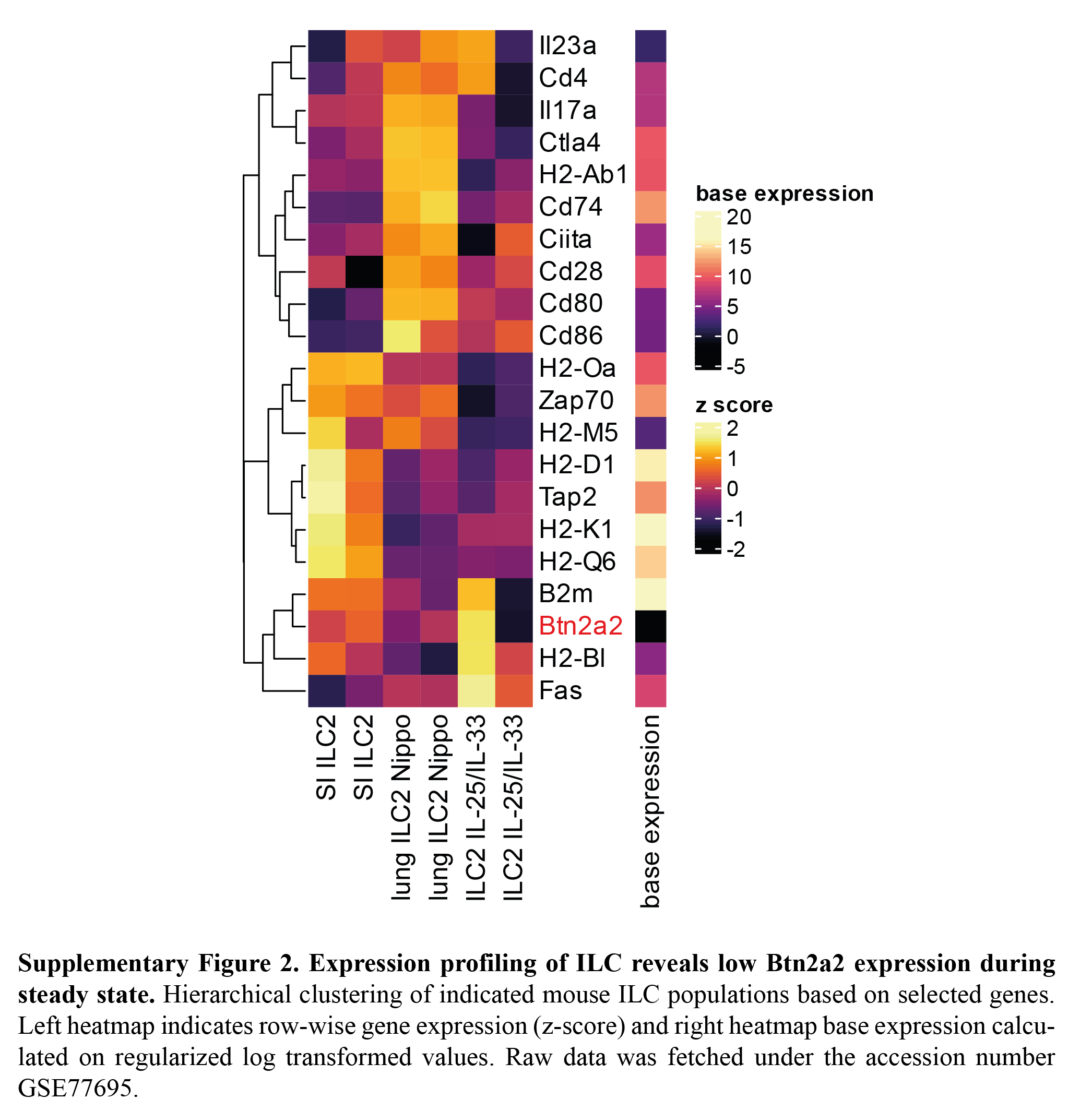


**Supplementary Table 1**. Genes that were found as markers for BTN2A2 expressing cells and have an average log2FC over BTN2A2 negative cells of ≥ 0.5 or ≤ -0.5.

| **Gene** | **p value** | **average log2FC** |
| --- | --- | --- |
| RP11-192H23.5 | 8,8E+10 | 1,19E+00 |
| BTN2A2 | 2,2E-127 | 1,06E+00 |
| MS4A1 | 1,7E-02 | 9,94E-01 |
| RNU4ATAC | 5,4E-02 | 9,50E-01 |
| CCL5 | 4,4E-01 | 9,20E-01 |
| RP11-81H14.2 | 7,7E+09 | 8,96E-01 |
| SPRY1 | 1,0E-03 | 8,68E-01 |
| U1..13 | 7,8E-01 | 8,57E-01 |
| U1..54 | 2,3E-02 | 8,41E-01 |
| GPAA1 | 8,4E+09 | 8,24E-01 |
| CST7 | 8,5E-01 | 8,09E-01 |
| MIR155HG | 2,7E-03 | 8,05E-01 |
| FAM54B..11 | 3,7E-02 | 7,96E-01 |
| GZMA | 9,3E-01 | 7,84E-01 |
| CD83 | 5,4E+11 | 7,25E-01 |
| CAPN1 | 7,6E-03 | 7,13E-01 |
| KLRF1 | 2,2E-01 | 7,13E-01 |
| J01415.20 | 1,5E-01 | 6,98E-01 |
| HLA-DRA | 1,4E-01 | 6,78E-01 |
| AE000661.37 | 1,4E-01 | 6,71E-01 |
| CHD3 | 8,0E-03 | 6,69E-01 |
| RNU12 | 9,6E-02 | 6,66E-01 |
| LDLRAD4..4 | 1,7E-01 | 6,43E-01 |
| C1orf123 | 2,8E-02 | 6,43E-01 |
| LEF1 | 2,2E-01 | 6,36E-01 |
| C12orf4 | 2,9E-03 | 6,24E-01 |
| U1..56 | 1,6E-01 | 6,19E-01 |
| ERCC-58.59375:mix1-87.890625:mix2..2 | 1,6E-03 | 6,19E-01 |
| DKC1 | 7,1E-01 | 6,04E-01 |
| NDUFS2 | 2,1E-03 | 5,95E-01 |
| RNU5A-1 | 5,5E-02 | 5,91E-01 |
| PSMA3 | 3,4E-03 | 5,87E-01 |
| U2..55 | 7,8E-02 | 5,84E-01 |
| GPS1..1 | 3,1E+11 | 5,78E-01 |
| C22orf25..2 | 1,1E-01 | 5,77E-01 |
| CKLF..1 | 3,3E-03 | 5,76E-01 |
| U2..37 | 7,4E-02 | 5,72E-01 |
| MCRS1 | 6,0E+11 | 5,72E-01 |
| VPRBP | 3,4E-02 | 5,65E-01 |
| C8orf59..1 | 1,9E+12 | 5,59E-01 |
| J01415.7 | 8,4E-03 | 5,58E-01 |
| ABHD5 | 5,4E-03 | 5,50E-01 |
| CDK11B | 4,4E-03 | 5,48E-01 |
| TRDJ3 | 5,0E-02 | 5,33E-01 |
| RILPL2 | 5,7E-02 | 5,31E-01 |
| PEF1 | 8,1E-03 | 5,30E-01 |
| TRBJ2-5 | 2,9E-01 | 5,28E-01 |
| NDUFAF5 | 1,2E-02 | 5,27E-01 |
| GON4L | 8,5E-03 | 5,25E-01 |
| CD58 | 2,3E-03 | 5,24E-01 |
| PPP6R3 | 4,2E+12 | 5,22E-01 |
| RP11-388M20.6 | 1,3E-02 | 5,22E-01 |
| J01415.19 | 2,1E-02 | 5,21E-01 |
| FUT8 | 2,3E+10 | 5,15E-01 |
| CCDC104 | 1,6E-02 | 5,12E-01 |
| LST1 | 1,6E-01 | 5,11E-01 |
| TGM2 | 1,0E-03 | 5,08E-01 |
| PPIE | 8,0E-01 | -5,06E-01 |
| RP11-58B17.1..1 | 8,6E-01 | -5,08E-01 |
| DPM3 | 2,1E-01 | -5,16E-01 |
| DUT | 6,2E-01 | -5,16E-01 |
| VKORC1..1 | 4,7E-02 | -5,17E-01 |
| TMED4 | 4,6E-02 | -5,17E-01 |
| GPN3 | 2,3E-01 | -5,21E-01 |
| ZBTB20 | 5,3E-02 | -5,23E-01 |
| BIRC3 | 9,3E-01 | -5,25E-01 |
| CYC1 | 2,4E-01 | -5,27E-01 |
| RGS1 | 5,7E-01 | -5,28E-01 |
| GPR183 | 4,1E-01 | -5,31E-01 |
| GPS2..1 | 9,0E-01 | -5,32E-01 |
| FCER1G | 4,7E-01 | -5,34E-01 |
| COX6A1 | 3,9E-02 | -5,39E-01 |
| DHRS7 | 8,4E-01 | -5,40E-01 |
| CYFIP2 | 1,7E-02 | -5,68E-01 |
| S100A13 | 3,5E-03 | -5,76E-01 |
| ARL4A | 3,0E-01 | -5,91E-01 |
| NEDD8 | 2,7E-01 | -5,96E-01 |
| PPOX | 1,9E-02 | -6,49E-01 |
| ERCC-58.59375:mix1-117.1875:mix2..1 | 1,5E-01 | -6,67E-01 |
| HERPUD1 | 4,5E-01 | -6,76E-01 |
| CLNS1A | 1,1E-01 | -6,78E-01 |
| ERCC-29.296875:mix1-7.32421875:mix2..2 | 3,3E-01 | -7,27E-01 |
| HOPX | 1,8E-01 | -7,28E-01 |
| BCKDHA..1 | 2,4E-01 | -7,45E-01 |
| SRIP3 | 8,6E-02 | -8,95E-01 |
| TMEM147 | 8,5E+10 | -9,77E-01 |


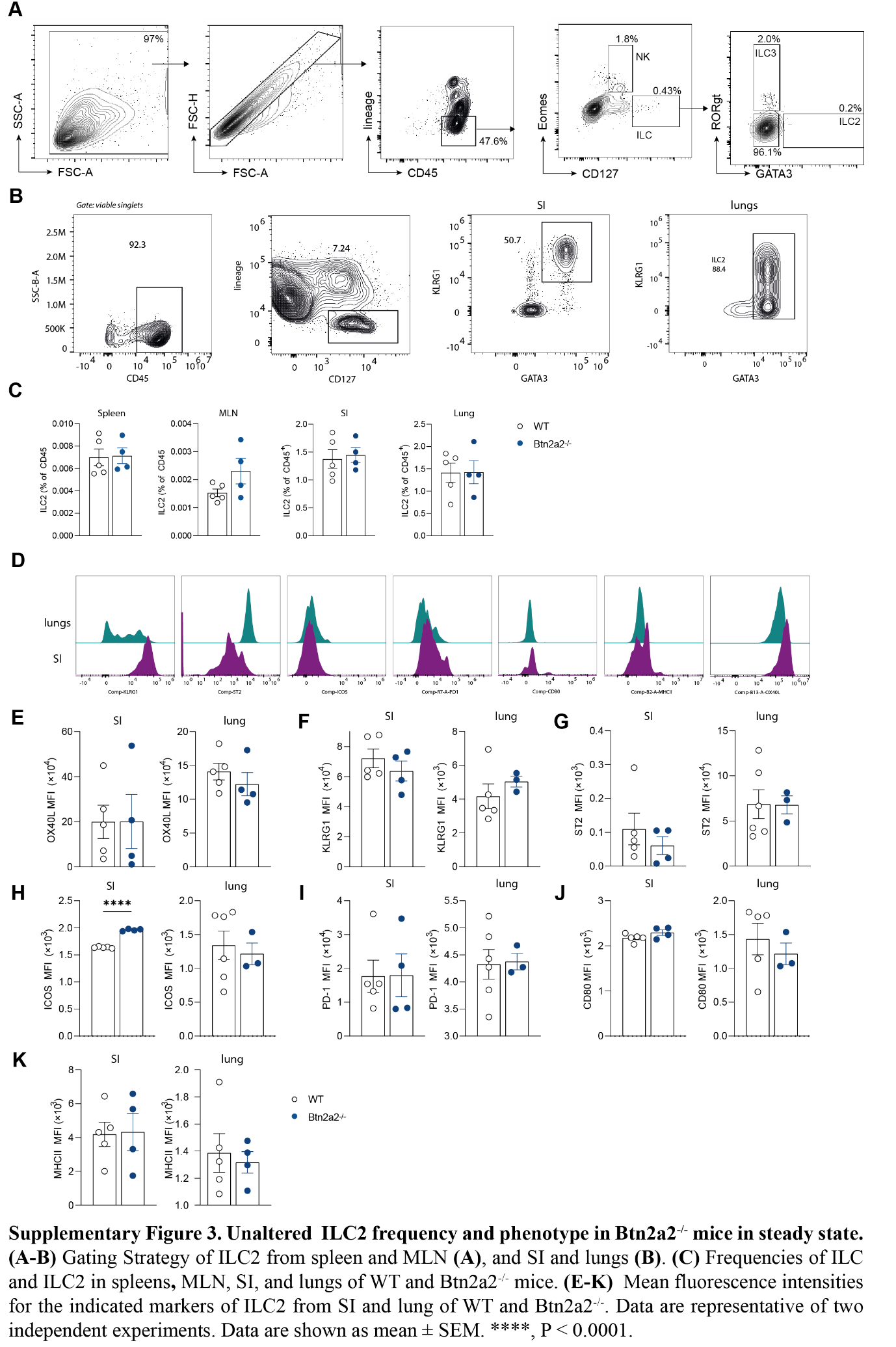


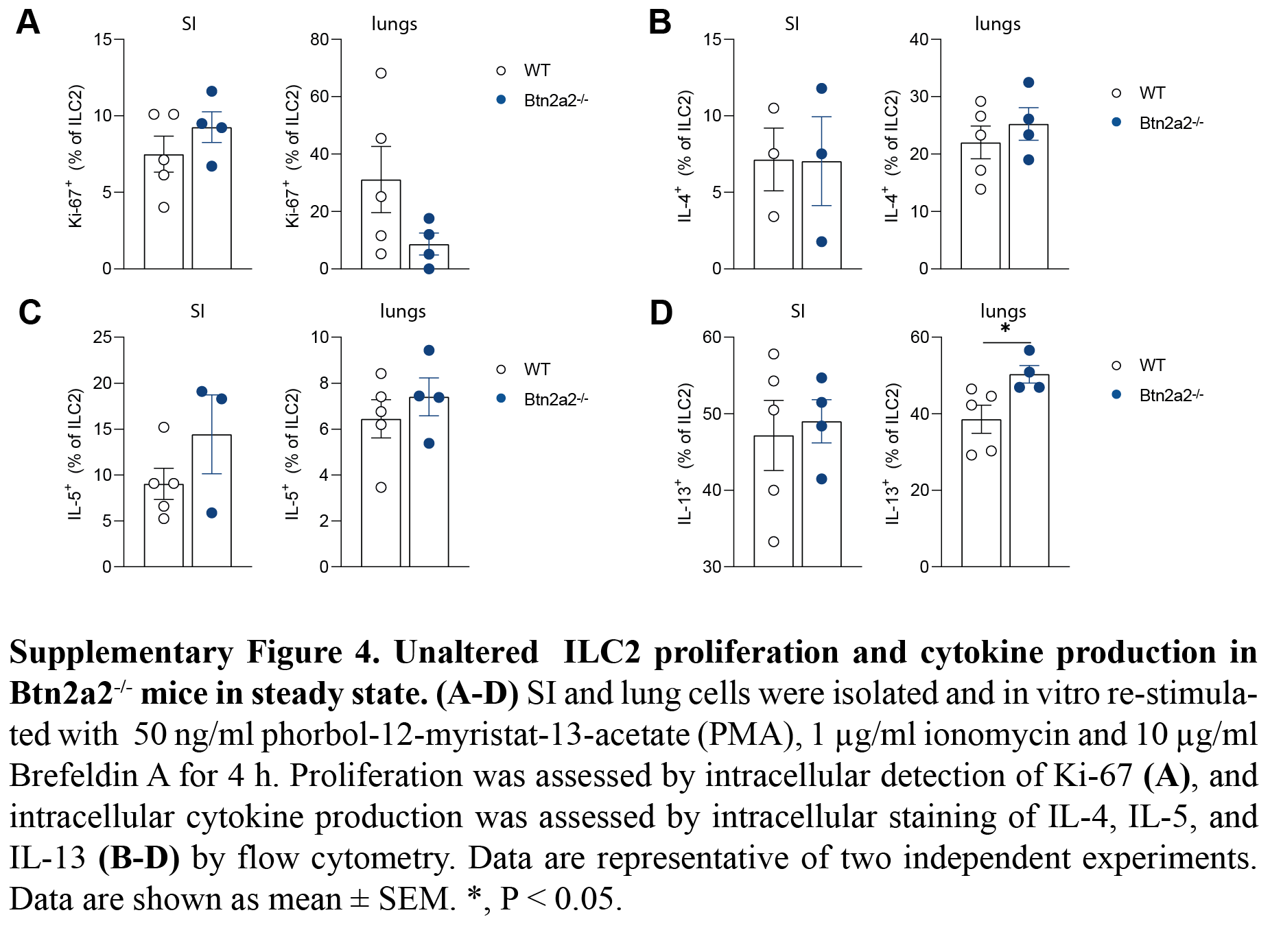


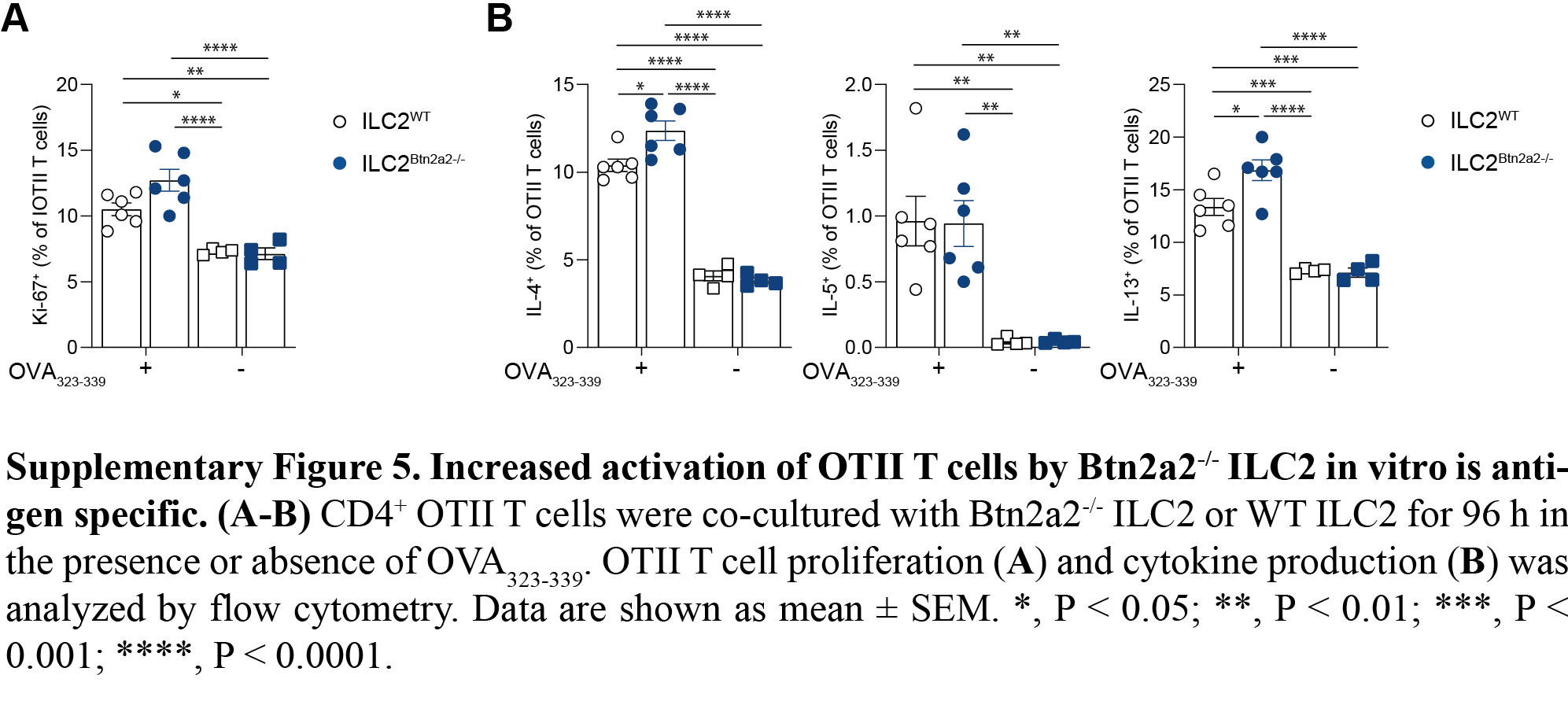


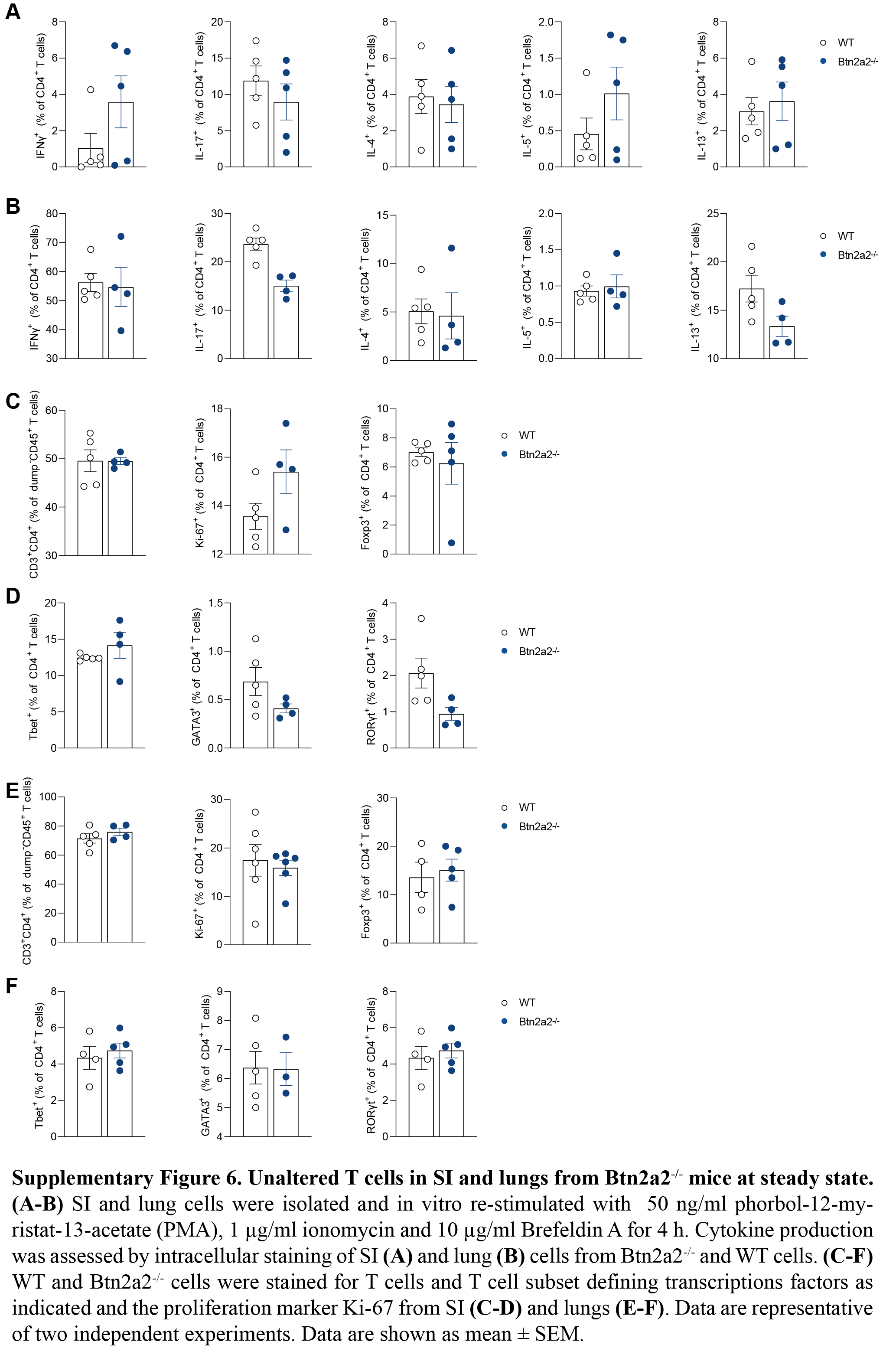


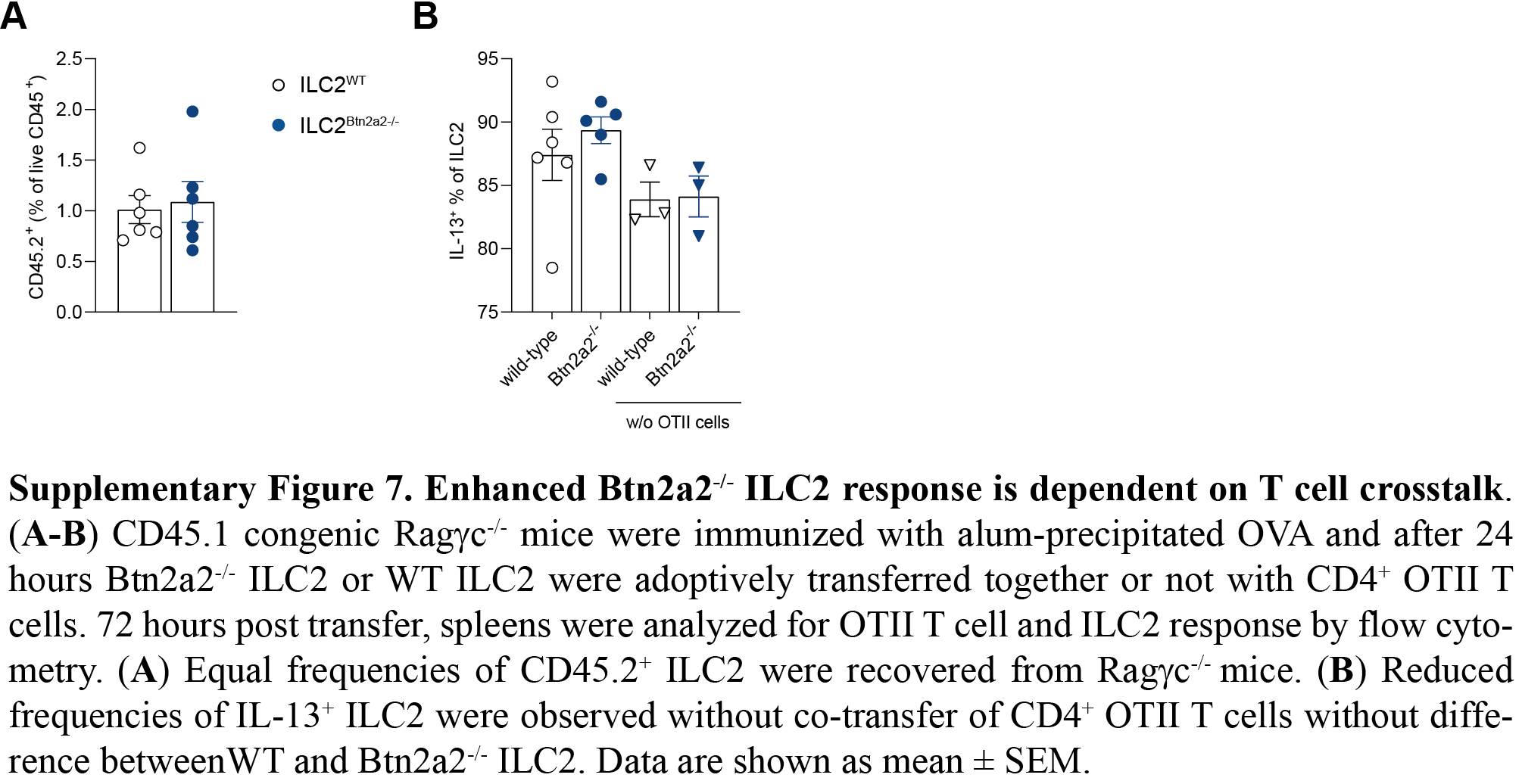

Supplement: Supplementary file 1 [file DataSheet_1.docx]
